# Fluorescence in situ hybridisation for interphase chromosomal aberration-based biological dosimetry

**DOI:** 10.1093/rpd/ncac264

**Published:** 2023-09-18

**Authors:** Prabodha Kumar Meher, Lovisa Lundholm, Andrzej Wojcik

**Affiliations:** Centre for Radiation Protection Research, Department of Molecular Biosciences, The Wenner-Gren Institute, Stockholm University, Stockholm, Sweden; Centre for Radiation Protection Research, Department of Molecular Biosciences, The Wenner-Gren Institute, Stockholm University, Stockholm, Sweden; Centre for Radiation Protection Research, Department of Molecular Biosciences, The Wenner-Gren Institute, Stockholm University, Stockholm, Sweden; Institute of Biology, Jan Kochanowski University, Kielce, Poland

## Abstract

Metaphase spreads stained with Giemsa or painted with chromosome-specific probes by fluorescence in situ hybridisation (FISH) have been in use since long for retrospective dose assessment (biological dosimetry). However, in cases of accidental exposure to ionising radiation, the culturing of lymphocytes to obtain metaphase chromosomes and analysis of chromosomal aberrations is time-consuming and problematic after high radiation doses. Similarly, analysing chromosomal damage in G0/G1 cells or nondividing cells by premature chromosome condensation is laborious. Following large-scale radiological emergencies, the time required for analysis is more important than precision of dose estimate. Painting of whole chromosomes using chromosome-specific probes in interphase nuclei by the FISH technique will eliminate the time required for cell culture and allow a fast dose estimate, provided that a meaningful dose-response can be obtained by scoring the number of chromosomal domains visible in interphase nuclei. In order to test the applicability of interphase FISH for quick biological dosimetry, whole blood from a healthy donor was irradiated with 8 Gy of gamma radiation. Irradiated whole blood was kept for 2 h at 37**°**C to allow DNA repair and thereafter processed for FISH with probes specific for Chromosomes-1 and 2. Damaged chromosomal fragments, distinguished by extra color domains, were observed in interphase nuclei of lymphocytes irradiated with 8 Gy. These fragments were efficiently detected and quantified by the FISH technique utilising both confocal and single plane fluorescence microscopy. Furthermore, a clear dose-response curve for interphase fragments was achieved following exposure to 0, 1, 2, 4 and 8 Gy of gamma radiation. These results demonstrate interphase FISH as a promising test for biodosimetry and for studying cytogenetic effects of radiation in nondividing cells.

## Introduction

Analysis of metaphase chromosomal aberrations has been a reliable and standard approach for biodosimetry and cytogenetics studies^(1–5)^. However, this method has several limitations in a scenario of mass scale casualty because of radiation accidents or nuclear threat. For instance, the standard dicentric assay-based biodosimetry requires several days^(3, 6, 7)^, also in the event of high dose exposure the radiation-induced cell cycle arrest and resulting low mitotic index makes this analysis challenging^(8)^. The metaphase staining approach using premature chromosome condensation (PCC) in G0 and nondividing cells has been reported to be useful for biodosimetry at high doses^(7, 9, 10)^. However, PCC requires high skills and is laborious^(11)^. In case of large-scale radiological emergencies, the time required for analysis is more important than precision of dose estimate^(1)^ and the alternative approaches for quick and reliable radiation biodosimetry are still needed^(3)^. In the present study, we investigated the applicability of the fluorescence in situ hybridisation (FISH) assay directly on whole-blood leukocytes to overcome the time required for culturing blood and skipping the challenging PCC-based biodosimetry. Interestingly, by applying FISH assay in interphase cells of gamma-irradiated, non-stimulated whole blood, we found a good gamma dose–response curve up to 8 Gy. The results suggest the interphase FISH test as an alternative or complementary biodosimetric tool to metaphase FISH or PCC. This assay may also serve as a convenient tool for studying chromosomal aberrations in nondividing G0/G1 cells.

## Materials and methods

### Collection of blood and irradiation

Blood samples were collected into heparinized tubes (Li-Heparin, 9 mL, Vacutest Kima, Italy) from a healthy donor, aliquoted 1 mL each in a 10 mL tube and irradiated within 3-h post-collection. Prior to irradiation, whole-blood samples were incubated for at least 20 min at 37°C in a water bath and irradiated at 37°C with 0, 1, 2, 4 and 8 Gy of ^137^Cs gamma radiation using Gammacell 40 Exactor (AECL, Canada) at a dose rate of 0.79 Gy/min. After irradiation, blood samples were transferred to the incubator at 37°C and kept for 2 h to allow DNA repair.

### Interphase FISH

Before proceeding for FISH, the whole blood in the tubes was processed for standard cytogenic slide preparation. In brief, whole blood was treated with hypotonic solution (75 mM KCl) at 37°C for 10 min, and washed several times with freshly prepared cold Carnoy’s fixative (3:1 methanol:acetic acid) until clear cell pellets were obtained. In the end, the clear cell pellets were diluted with few drops of fixative and 20 μL of cell suspension was dropped on a clean microscopic slide. The slides were kept at room temperature for few hours to air dry. Thereafter, slides were processed for the FISH assay following the manufacturer’s protocol using whole-chromosome paint probes for Chromosomes-1 and 2 (XCP 1 Orange, XCP 2 Green; XCyting Chromosome Paints, MetaSystems GmbH, Germany). The slides were scanned using a fluorescent microscope with ×100 oil immersion lens (Nikon Eclipse E800, Nikon, Tokyo, Japan), images were acquired with auto exposure setting using a Cool Cube 1 CCD camera and the image analysis system ISIS (MetaSystems GmbH, Germany). AHH-1 lymphoblast cells were cultured in RPMI-1640 medium with 25 mM HEPES (Sigma-Aldrich), supplemented with 10% bovine calf serum (HyClone), 1% penicillin–streptomycin, 1% L-glutamine (200 mM) and 1% sodium pyruvate solution (100 mM), all from Sigma-Aldrich. Cells were grown at 37°C and 5% CO_2_. Exponentially growing AHH-1 cells were fixed and stained with the same protocol as blood. Fifty images for mononuclear cell were scored per dose point and the extra color domains were counted by looking at both merged images and also at individual channel exposure image using the ISIS software. The Poisson distribution, 95% lower confidence limit (LCL), 95% upper confidence limit (UCL) and index of dispersion were calculated using the NETA 1.0 software as described elsewhere^(12)^.

### Statistical analysis

The frequency of 8 Gy induced aberrations detected by interphase FISH was compared with the control value by the two-sided Student’s *t*-test. A *p*-value <  0.05 was considered significant.

## Results

### Regular color domains identified by FISH in interphase nuclei

**Figure 1:**
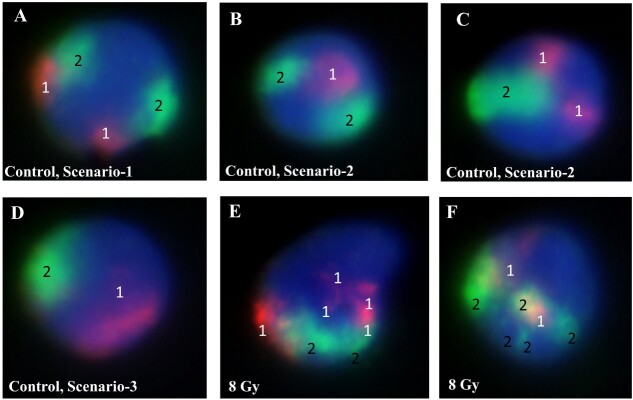
Representative images of interphase FISH in mononuclear cells. Human Chromosome-1 in orange color (marked with 1), Chromosome-2 in green color domain (marked with 2), nuclei in blue color. (**A**) Nonirradiated cell, Scenario-1; four color domains are seen, i.e. both chromosome pairs are easily distinguished. (**B**) Nonirradiated cell, Scenario-2; three color domains are seen, Chromosome-1 in orange is overlapped. (**C**) Nonirradiated cell, Scenario-2; three color domains are seen, Chromosome-2 in green is overlapped. (**D**) Nonirradiated cell, Scenario-3; two color domains are seen, Chromosome-1 pair in orange is overlapped, and Chromosome-2 pair in green is overlapped. (**E**, **F**) Mononuclear cell irradiated with 8 Gy post 2 h of incubation, extra color domains are seen for both Chromosome-1 in orange and Chromosome-2 in green.

The FISH assay was carried out with chromosome paint probes for two chromosomes having two colors (Chromosome-1 in orange; Chromosome-2 in green) and nuclei were stained with DAPI. Hence, in a two-dimensional (2D) FISH image of mononuclear cell nuclei (from nonirradiated blood), the Chromosome-1 pair should produce two distinguishable orange color domains and the Chromosome-2 pair should be seen as two distinguishable green color domains. This outcome is possible when neither of the chromosomes are overlapping. This scenario, categorised as Scenario-1, is shown in Figure 1A. Scenario-2 describes the situation when only three distinguished color domains are seen because two homologous domains of one chromosome overlap (Figure 1B and C). Scenario-3 describes the situation when both homologous domains overlap resulting in two distinguished color domains (Figure 1D). There could also be a rare situation that can be considered as Scenario-4, where all chromosome pairs are exactly overlapped. However, we have not observed this scenario during our scoring. Based on the observations and appearance of distinguished color domains for the probed chromosome pairs in nonirradiated whole blood, we defined possible criteria for identifying chromosomal aberrations (breaks/translocations) induced by radiation. We assumed that an excess of two distinguished color domains for each color would represent breaks/translocations. Consequently, the scoring of aberrations should be restricted to nuclei having at least four-color domains (i.e. Scenario-1) and to nuclei having more than two distinguishable single-color domain and at least one domain of another color (i.e. Scenario-2). In order to reduce ambiguity, nuclei corresponding to the other scenarios should be excluded from scoring.

### Radiation-induced excess color domains and chromosomal translocation identified by FISH in interphase nuclei

**Figure 2:**
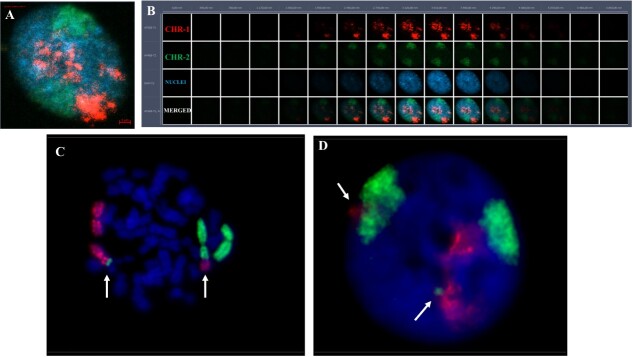
Radiation-induced extra color domains in interphase nuclei are breaks/translocations. (**A**) 2D confocal microscopy images of 8 Gy irradiated mononuclear cells, nuclei in blue, Chromosome-1 in orange, Chromosome-2 in green color, all merged. Extra color domains for Chromosome-1 in orange are clearly seen. (**B**) Different Z-stack acquisitions of images of the same nuclei, showing acquisition of individual channel and also merged. The extra color domains are found to be clear breaks/translocations (**C**) Metaphase FISH of nonirradiated AHH-1 cell showing stable translocations between Chromosome-1 in orange and Chromosome-2 in green (indicated by white arrows). (**D**) Interphase FISH image of nonirradiated AHH-1 cell, where the stable translocations are seen as extra color domains (indicated by white arrows) in interphase nuclei.

**Figure 3:**
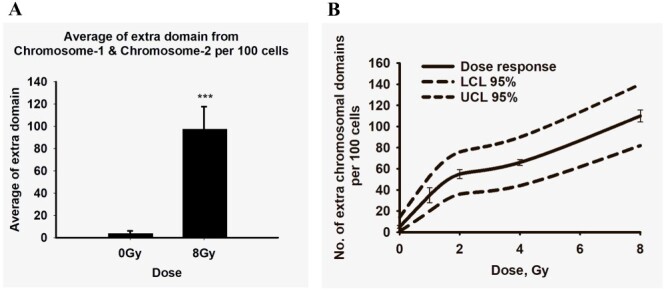
Quantification of extra color domains from interphase FISH and dose-response curve in whole blood. (**A**) Significant increase in average extra color domains (involving Chromosomes-1 and 2) were observed in the whole-blood samples (8 Gy of gamma radiation at 2-h postexposure) compared with 0 Gy samples (*n* = 3, two-tailed *p*-value = 0.0013). (**B**) Dose–response curve for the interphase FISH (*n* = 2) displaying average values as well as the 95% LCL and UCL. Error bars represent standard deviation. The frequency of extra color domain (representing breaks/translocations) is also found to be Poisson distributed.

In order to test the scoring criteria, we irradiated a whole-blood sample with 8 Gy of gamma radiation, incubated the cells for 2 h at 37°C and processed for FISH along with a control sample. Fifty mononuclear cells were scored from Scenarios-1 and 2 images for each treatment point. In the irradiated cells, we observed clearly distinguishable extra color domains (excess of two domains for each chromosome) for both chromosome pairs (Figure 1E and F). The dose of 8 Gy induced a significant elevation of the excess color domains above the control level (Figure 3A). A possibility existed that the excess color domains did not represent domain discontinuities (resulting from chromosomal aberrations), but resulted from elongated and crooked domains, parts of which were out of focus and not visible on 2D images (representing intact domains). In order to verify that this is not the case, we analysed several irradiated cells with confocal microscopy (LSM 800, Carl Zeiss Microscopy GmbH, Germany) using Z-stacking (Figure 2A and B) and 3D rotation view (a video can be seen at the link https://youtu.be/Y83ZHMMPHbg). The observations support the idea that the extra chromosome domains represent domain discontinuities resulting from chromosomal breaks or interchromosomal translocations. In addition, as a positive control, we carried out FISH on nuclei of nonirradiated AHH-1 cells that have a stable translocation between Chromosomes-1 and 2 (Figure 2C). These cells were acquired from ATCC (USA) and are routinely used in our laboratory. Extra color domains for this translocation could be identified in interphase nuclei (Figure 2D) from the 2D images obtained through normal fluorescence microscope. Collectively, these observations suggest that extra color domains seen in 2D images represent chromosomal aberrations.

### Dose-response curve for excess color domains

A dose-response curve for the excess color domains was setup in order to validate the applicability of the test for the purpose of retrospective biological dosimetry. Two independent experiments were carried out with blood of one donor. The values from each experiment of excess color domains, 95% LCL and UCL, indices of dispersion, mean of extra chromosomal breaks (ECB) and standard deviation from mean are given in Table 1. The frequencies of extra color domains were also found to be Poisson distributed (Table 1). A clear dose-response curve for gamma doses of 0, 1, 2, 4 and 8 Gy was achieved (Figure 3B). The frequencies of extra color domains were also found to be Poisson distributed (Table 1). It was interesting to compare the obtained dose-response to dose-response curves for PCC fragments and excess chromosomal breaks in metaphase chromosomes of human peripheral blood lymphocytes exposed to gamma radiation. To this end, dose-response curves for PCC fragments observed by Pantelias *et al*.^(10)^ in cells fused 2 and 48 h after gamma radiation exposure and genomic translocations after 76 h by Amula *et al*.^(13)^ were plotted on the same graph as extra color domains. The results are shown in supplementary data (Figure S1). The PCC fragments were scored in Giemsa-stained cells and the genomic translocations observed were with FISH assay. In order to make the results compatible with the extra color domains, the frequencies of PCC and chromosome fragments were multiplied by 0.294, which corresponds to the fraction of the genome covered by Chromosomes-1 and 2^(14)^.

**Table 1:** Number of extra chromosomal domains (involving Chromosomes-1 and 2) per 100 nuclei in each dose-response curve. For each dose, 50 cells (with Scenarios-1 and 2) were manually scored and data represented here are for 100 nuclei.

Dose (Gy)	Number of extra chromosomal domains (Chromosomes-1 and 2) per 100 nuclei									
	Dose-Response-1				Dose-Response-2				Mean (ECB)	SD
	ECB	LCL	UCL	Index of dispersion	ECB	LCL	UCL	Index of dispersion		
0	4	0.8	14	0.97	6	0.8	14	0.95	5	1.41
1	30	16	48	0.71	40	24	58	0.92	35	7.07
2	52	34	72	0.80	58	38	80	1.13	55	4.24
4	64	42	88	1.38	68	46	92	0.93	66	2.82
8	114	86	114	1.03	106	78	136	0.82	110	5.65

## Discussion

Our results demonstrate that the interphase FISH assay allows to identify and quantify extra color domains that reflect chromosomal aberrations generated by exposure to gamma radiation. The clearly distinguishable chromosomal domains observed by us reflect the fact that chromosomes occupy distinct domains in nuclei of somatic cells^(15, 16)^. We analysed 50 cells on 2D images by using whole-chromosome probes for Chromosome-1 in orange and Chromosome-2 in green. A major problem was the potential overlapping or co-localisation of homologous chromosome domains that could lead to missed aberrations^(17)^. We addressed this by categorising the possible scenarios of chromosomal domains in 2D images and restricting scoring to Scenario-1 where both homologous domains were distinguishable and Scenario-2 where two homologous domains of one chromosome are distinguishable and the other two domains overlap. The correctness of this approach was demonstrated by the clear dose-response curve for gamma radiation up to 8 Gy. However, we cannot rule out that our exclusion criteria produced minor alterations in the response estimation. The shape of the dose-response may vary from lab to lab depending on the expertise in FISH hybridisation staining methods, quality of images taken and individual scoring criteria.

The simplicity of the scoring procedure suggests that the assay is suitable for automation. It has been suggested that the automation of image examination and analysis of chromosomal aberration would aid in speeding up the estimation of dose and better support medical management of exposed individuals^(4, 5, 18)^. This option has not yet been tested by us but is envisaged as a future project. To eliminate the possibility of false-positive identification of aberrations, the extra color domains were reconfirmed as distinguished chromosomal discontinuities by looking at the interphase FISH nuclei in different Z-stacks under confocal microscopy. Moreover, a positive control was analysed in the form of AHH-1 cells that exhibit a stable, reciprocal translocation between Chromosomes-1 and 2. The reciprocal translocation could clearly be identified as two extra color spots in the interphase nuclei. This finding demonstrates that interphase FISH can be implemented to study chromosomal aberrations in nuclei of cells that are arrested in the cell cycle or that poorly divide. More experiments are needed to confirm the broad applicability of this approach. With respect to biological dosimetry, a suitable application could be for accidents involving very high doses, where the proliferation of lymphocytes is inhibited^(11, 19)^. It has been shown that distributions of chromosomal aberrations induced by X-rays or gamma radiation exposure follow a Poisson distribution^(20)^. We analysed the distributions of extra chromosomal domains and found that also they are Poisson distributed. This finding is important because it opens the possibility of extracting information on whether the radiation exposure of the blood donor was whole-, or partial body^(21)^. More experiments are needed to assess the sensitivity of the assay to the degree of dose inhomogeneity.

Finally, it was interesting to compare the steepness of the dose-response for extra chromosomal domains with dose-responses of PCC fragments and translocations reported by other authors. The interesting question is whether the extra color domains detected by interphase FISH represent both fragments and chromosomal exchanges. In general, the PCC fragment numbers are higher shortly postexposure and gradually decline with time postexposure^(22)^. Chromosomal exchanges associated with joining of primary PCC breaks were shown to increase with time postexposure^(23)^. The fact that the frequency of extra color domains detected 2-h postexposure is so much lower than the number of PCC fragments detected after the same time suggests that the methods primarily detect translocations. It is possible that breaks are not detected because the acentric fragments remain inside a chromosomal domain and are only detectable on metaphase spreads. Nevertheless, some fragments appear to be shuttled out of the domains because the frequency of extra color domains is higher than that of PCC fragments and translocations detected after 48-h postexposure. High-resolution imaging could perhaps provide additional information to this issue.

In summary, we demonstrated that the interphase FISH assay can be a suitable extension of traditional FISH for overcoming the time required by culturing the blood for the metaphase FISH-based biodosimetry. Furthermore, this assay can also be a convenient tool in studying chromosomal aberrations in nondividing and G0/G1 phase cells.

## Supplementary Material

Supplimentary_Figure_MS_Interphase_FISH_ncac264

## Data Availability

The data that support the findings (cell images) of this study are available on request from the corresponding author, [PKM].
