# Supplementary material for: Fluorescence in situ hybridisation for interphase chromosomal aberration-based biological dosimetry
Source: Radiat Prot Dosimetry. 2023 Sep 18;199(14):1501–7. doi: 10.1093/rpd/ncac264 (PMC10505941; doi:10.1093/rpd/ncac264)
Supplement: Supplimentary_Figure_MS_Interphase_FISH_ncac264 [file supplimentary_figure_ms_interphase_fish_ncac264.docx]

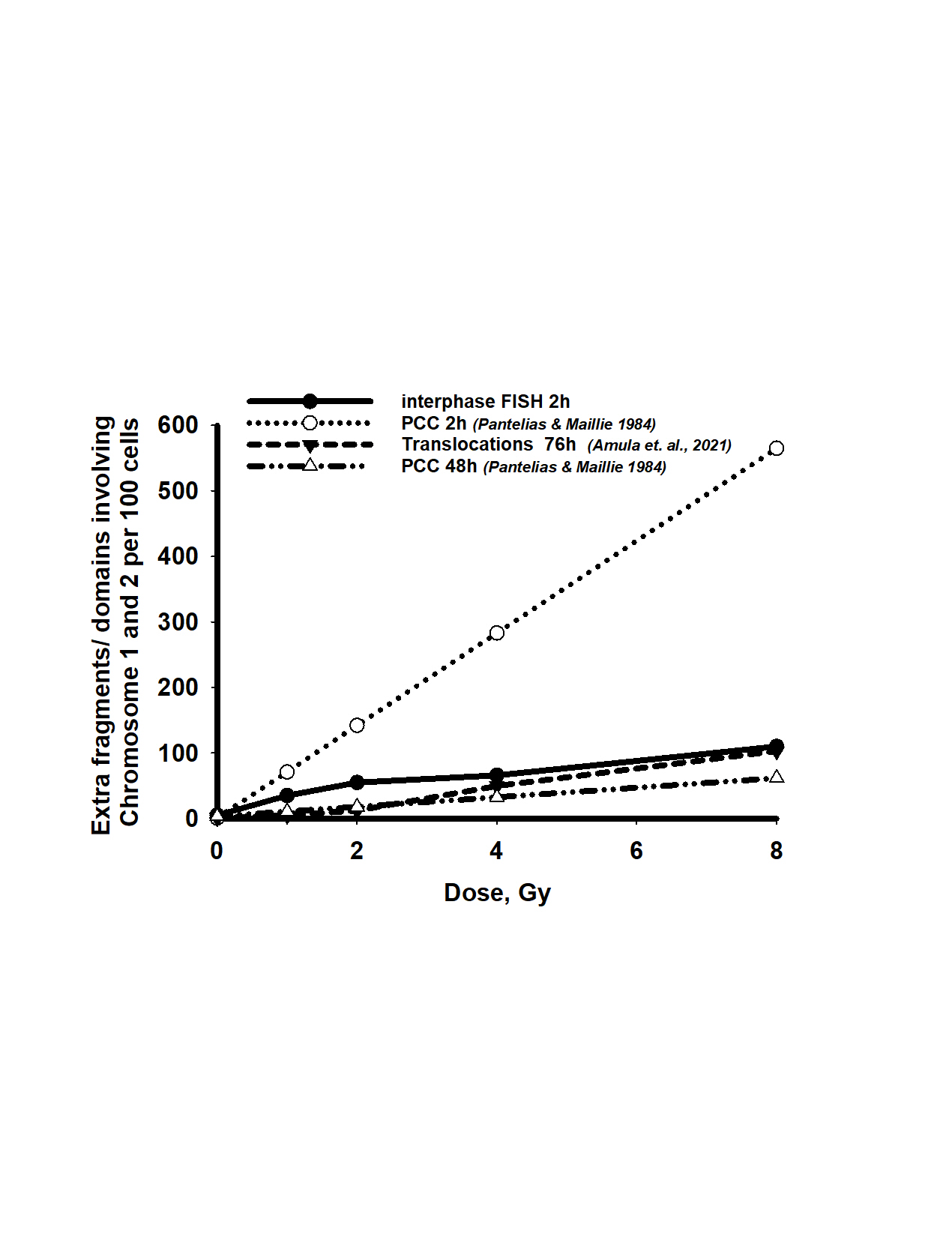


Figure S1. Comparison of dose response curves. Interphase FISH 2h dose response curve is from the present study. PCC & translocation dose responses were adopted and replotted by scaling to chromosome 1 and chromosome 2 (genomic conversion factor 0.294) from Pantelias & Maillie 1984 and Amula et. al., 2021, respectively.
